# ASC-Mediated Inflammation and Pyroptosis Attenuates *Brucella abortus* Pathogenesis Following the Recognition of gDNA

**DOI:** 10.3390/pathogens9121008

**Published:** 2020-11-30

**Authors:** Juselyn D. Tupik, Sheryl L. Coutermarsh-Ott, Angela H. Benton, Kellie A. King, Hanna D. Kiryluk, Clayton C. Caswell, Irving C. Allen

**Affiliations:** 1 Department of Biomedical Sciences and Pathobiology, Virginia-Maryland College of Veterinary Medicine, Virginia Tech, Blacksburg, VA 24061, USA; jdtupik@vt.edu (J.D.T.); slc2003@vt.edu (S.L.C.-O.); ahbenton@vt.edu (A.H.B.); kellieking@vt.edu (K.A.K.); hanna98@vt.edu (H.D.K.); caswellc@vt.edu (C.C.C.); 2 Department of Basic Science Education, Virginia Tech Carilion School of Medicine, Roanoke, VA 24016, USA

**Keywords:** brucellosis, canonical inflammasome, non-canonical inflammasome, NLR, pyroptosis, ASC, caspase-11, caspase-1, IL-1β, gDNA

## Abstract

*Brucella abortus* is a zoonotic pathogen that causes brucellosis. Because of *Brucella’s* unique LPS layer and intracellular localization predominately within macrophages, it can often evade immune detection. However, pattern recognition receptors are capable of sensing *Brucella* pathogen-associated molecular patterns (PAMPS). For example, NOD-like receptors (NLRs) can form a multi-protein inflammasome complex to attenuate *Brucella* pathogenesis. The inflammasome activates IL-1β and IL-18 to drive immune cell recruitment. Alternatively, inflammasome activation also initiates inflammatory cell death, termed pyroptosis, which augments bacteria clearance. In this report, we assess canonical and non-canonical inflammasome activation following *B. abortus* infection. We conducted in vivo studies using *Asc^−/−^* mice and observed decreased mouse survival, immune cell recruitment, and increased bacteria load. We also conducted studies with *Caspase-11^−/−^* mice and did not observe any significant impact on *B. abortus* pathogenesis. Through mechanistic studies using *Asc^−/−^* macrophages, our data suggests that the protective role of ASC may result from the induction of pyroptosis through a gasdermin D-dependent mechanism in macrophages. Additionally, we show that the recognition of *Brucella* is facilitated by sensing the PAMP gDNA rather than the less immunogenic LPS. Together, these results refine our understanding of the role that inflammasome activation and pyroptosis plays during brucellosis.

## 1. Introduction

Brucellosis is a zoonotic bacterial disease that exhibits pathogenesis consistent with inflammation. Transmitted through *Brucella* spp. primarily from agricultural animals to humans in unpasteurized dairy products, brucellosis symptoms in humans often include inflammatory or influenza-like characteristics such as arthritis, undulant fevers, and neurological manifestations [1,2]. Because there is currently no human vaccine for brucellosis and effective antibiotic regiments for the disease require long treatment durations, these symptoms often persist throughout the infected individual’s lifetime due to the well-adapted ability of *Brucella* to evade immune recognition [3]. Unlike the classical lipopolysaccharide (LPS) layer of Gram-negative bacteria such as *Escherichia coli* that contain a glucosamine backbone with short acyl groups, *Brucella* spp. contain a modified lipid A layer that consists of a diaminoglucose backbone with long branching acyl groups [2]. This deviation from a consistent molecular structure has the potential to subvert immune recognition by the innate immune system through complement interference and decreased cytokine production, leading to enhanced *Brucella* replication and pathogenesis. Despite its mechanisms of immune avoidance, there are some aspects of *Brucella* spp. that are recognized by the innate immune system, making the understanding of these mechanisms essential for targeting future treatments for brucellosis.

As hypothesized by Janeway (1989), the innate immune system has evolved over time to recognize consistent molecular structures in pathogens known as Pathogen or Damage-Associated Molecular Patterns (PAMPs or DAMPs). These PAMPs and DAMPs are recognized by protein structures known as pattern recognition receptors (PRRs) [4]. From previous studies, *Brucella* genomic DNA (gDNA) is known to be recognized by the PRR absent in melanoma 2 (AIM2) and subsequently promotes inflammation, making it an excellent PAMP for immune recognition [5,6,7]. PRRs include membrane-bound receptors, which consist of Toll-like receptors (TLRs) and C-type Lectin receptors (CLRs), as well as cytosolic receptors made up of a Nucleotide-Binding Domain and Leucine-Rich Repeat Containing receptors (NLRs), Aim-2-Like receptors (ALRs), Rig-I-Like Helicase receptors (RLRs), and the X-LR class of uncategorized receptors [8,9]. After the recognition of a PAMP or DAMP, PRRs generally serve as scaffolding proteins to promote the initiation or inhibition of immune signaling pathways [9]. Of the PRRs that have been described in brucellosis, the best characterized have been the TLRs. From previous literature, many TLRs have been implicated with *Brucella* detection, which plays a role in bacterial signaling, host resistance, and dendritic cell activation [10,11,12,13,14,15,16]. TLRs also play important roles in the transcriptional generation of inactive inflammatory cytokines in response to *Brucella* infections that can be activated by NLR or ALR immune signaling complexes [17,18]. This indicates that multiple PRRs work in tandem to attenuate brucellosis pathogenesis.

Inflammasome-forming NLRs and AIM2 have also been reported to play a role in *Brucella* sensing [5,6,7]. After recognition, the NLR or ALR is able to bind the apoptosis-associated speck-like protein containing a caspase activation recruitment domain (CARD) (ASC) and procaspase-1 to form the canonical inflammasome [8]. The inflammasome then cleaves caspase-1, which subsequently cleaves the cytokines pro-IL-1β and pro-IL-18, produced through TLR signaling, to their active forms to promote inflammation [8,9,19,20,21,22,23]. Inflammasome signaling can also lead to a form of inflammatory cell death known as pyroptosis. Pyroptosis occurs when activated capase-1 cleaves the protein gasdermin D, releasing the gasdermin N subunit [24]. This subunit binds with phosphoinositides on the cell membrane and oligomerizes, creating membrane pores that lead to an osmotic imbalance in the cell that eventually leads to cell lysis [24]. Recently, the formation of a non-canonical inflammasome has been described that utilizes caspase-11 to mediate the cleavage of gasdermin D to initiate pyroptosis.

Previous studies evaluating inflammasome activation in response to *Brucella* have predominately focused on characterizing the activation of inflammatory cytokine signaling associated with canonical inflammasome activation. The best described inflammasomes involved in *Brucella* infections are NLR Family Pyrin Domain Containing 3 (NLRP3) and AIM2. In mouse models, the NLRP3 inflammasome promotes survival and decreased bacterial load through enhanced cytokine secretion, in addition to sensing mitochondrial reactive oxygen species (ROS) generated from *Brucella* [6,7]. Looking at the *Brucella* PAMPs, AIM2, as a known sensor of bacterial DNA, becomes activated from *Brucella* gDNA recognition and initiates inflammatory cytokine signaling and pyroptosis [5,6,25]. These inflammasomes are ASC-dependent, as shown in the formation of punctate ASC structures during infection [6], indicating that ASC-dependent inflammasomes are important in *Brucella* recognition and targeting through inflammatory cytokine signaling. Despite these advancements in studying inflammasome-mediated inflammatory cytokine signaling, pyroptosis and the role of gasdermin D have not been extensively evaluated in response to *Brucella.*

In this study, we used *Asc^−/−^* and *Caspase-11^−/−^* mice to further elucidate the role of the canonical and non-canonical inflammasomes following *B. abortus* infection. We sought to assess survival, histopathology, bacterial load, and cell death to provide a more holistic view of cytokine responses and pyroptosis, both in vivo and *in vitro*. Additionally, we reassessed *Brucella* PAMPs using *B. abortus* gDNA and LPS to better define the mechanisms associated with pathogen recognition. Ultimately, we found that ASC functions to attenuate *B. abortus* pathogenesis through the modulation of inflammation and pyroptosis, requiring gasdermin D through a mechanism independent of caspase-11. Additionally, we determined that *Brucella* gDNA, rather than LPS, provoked an elevated inflammasome response that augmented pyroptosis. This report contributes to the current literature and provides some additional novel insights into potential mechanisms of inflammasome activation during brucellosis.

## 2. Results

### 2.1. ASC Attenuates B. abortus Pathogenesis and Is Critical for Host Survival

**Figure 1:**
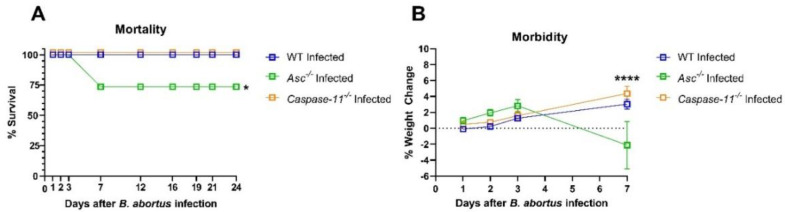
*Asc^−/−^* and *Caspase-11^−/−^* mortality and morbidity. *Asc^−/−^* (*n* = 19), *Caspase-11^−/−^* (*n* = 19), and C57BL/6 WT (*n* = 26) mice were injected intraperitoneally with 1 × 10^5^ *B. abortus* CFUs and assessed daily for excessive weight loss (>20%) warranting euthanasia according to the Institutional Animal Care and Use Committee (IACUC). (**A**) Mortality graph based on (**B**) a morbidity assessment of *Asc^−/−^*, *Capsase-11^−/−^*, and WT mice. All the mice were weighed for a 24-day period, with morbidity warranting euthanasia in *Asc^−/−^* mice only at Day 7. * *p* < 0.05, **** *p* < 0.0001.

To explore canonical and non-canonical inflammasome activation to *Brucella abortus*, we used mice that lack either the ASC adaptor protein (*Asc^−/−^)* or the non-canonical inflammasome-associated caspase, caspase-11 (*Caspase-11^−/−^*). After intraperitoneally injecting mice with 1 × 10^5^ colony forming units (CFUs) of *B. abortus*, we monitored mortality in all mouse groups for a 24-day period (Figure 1A). At Day 7, there was a 26.3% decrease in the survival of the *Asc^−/−^* mice. In these animals, the weight loss in 5 of the 19 mice exceeded 20%, and several of the *Asc^−/−^* mice developed clinical parameters associated with disease progression, such as decreased body condition, that required euthanasia (Figure 1B). However, there was no decrease in survival for the wildtype (WT) and *Caspase-11^−/−^* mice. These mortality data suggest that the canonical inflammasome plays a more critical role in host survival compared to the non-canonical inflammasome and caspase-11.

### 2.2. ASC Contributes to Inflammatory Pathogenesis during B. abortus Infection

**Figure 2:**
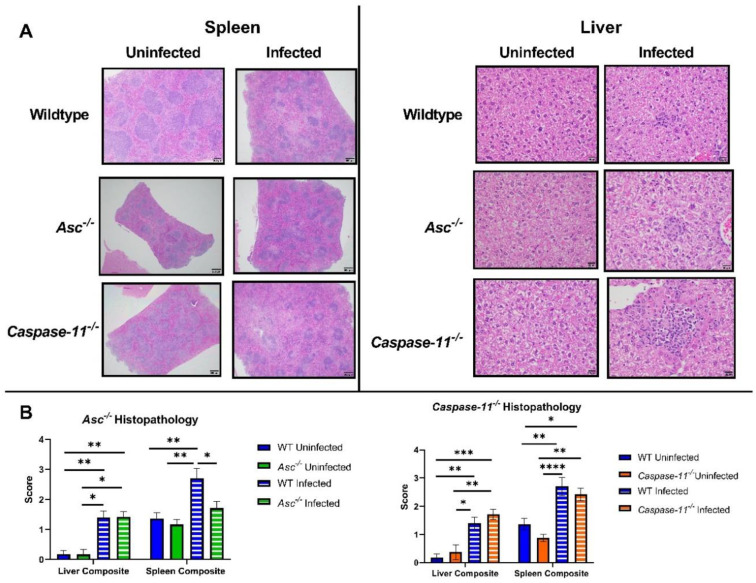
Inflammation in the liver and spleen. A total of 20 *Asc^−/−^* (*n =* 6 uninfected [U], 14 infected [I]), 27 *Caspase-11^−/−^* (*n* = 8 U, 19 I), and 34 C57BL/6 WT (*n* = 11 U, 23 I) livers and spleens were evaluated by histopathology 3 d.p.i. (**A**) H&E stained histological slides of the liver and spleen from WT, *Asc^−/−^*, and *Caspase-11^−/−^* mice. All spleen images were taken at 4× power and all liver images were taken at 40× power. Inflammation and extramedullary hematopoiesis (EMH) were the dominant features observed in the histopathology evaluation. (**B**) Bar graphs of WT, *Asc^−/−^*, and *Caspase-11^−/−^* histopathology composite scores were generated based on inflammation and EMH in the liver and spleen. * *p* < 0.05, ** *p* < 0.01, *** *p* < 0.001, **** *p* < 0.0001.

To further elucidate the role of ASC and caspase-11 activation *in vivo*, we conducted a histopathological analysis on the liver and spleen from wildtype, *Asc^−/−^*, and *Caspase-11^−/−^* mice three days post-infection. Histopathology indicated that all the infected mouse groups exhibited elevated extramedullary hematopoiesis (EMH) and inflammation in the liver and spleen (Figure 2A,B).

Wildtype mice exhibited higher EMH and inflammation scores compared to the *Asc^−/−^* animals in the spleen. This trend was not observed in the *Caspase-11^−/−^* mice in either the liver or the spleen. Together, these data suggest that ASC augments splenic inflammation and plays a key role in *B. abortus*-mediated pathology in the spleen, which is a target organ in this mouse model. The failure to mount a vigorous immune response to *B. abortus* in the *Asc^−/−^* mice is likely associated with the increased morbidity and mortality.

### 2.3. Bacterial Load Is Significantly Increased in the Absence of ASC and Decreased in the Absence of Caspase-11

**Figure 3:**
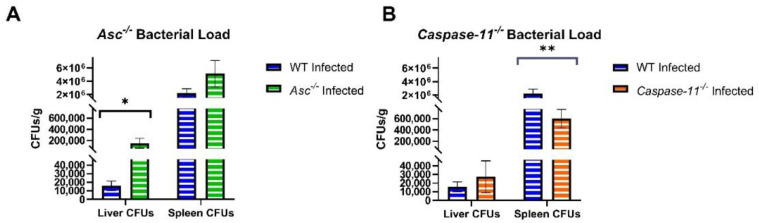
*Brucella* CFUs in the liver and spleen of *Asc^−/−^* and *Caspase-11^−/−^* mice. Three d.p.i. liver and spleens from infected C57BL/6 WT (*n* = 23), **(A)** *Asc^−/−^* (*n* = 14), and **(B)** *Caspase-11^−/−^* (*n* = 19) mice were homogenized and counted for CFUs/gram. * *p* < 0.05, ** *p* < 0.01.

Bacterial loads were determined in the spleen and liver of *B. abortus*-infected animals 3 d.p.i (Figure 3). Between wildtype, *Asc^−/−^*, and *Caspase-11^−/−^* mice, there was no significant difference in the weight of the spleens used for analysis.

In *Asc^−/−^* mice, we observed significantly elevated *B. abortus* CFUs in the liver and a trending increase in the spleen. In the *Caspase-11^−/−^* mice, we observed a similar trending increase in CFUs in the liver. However, in the spleen *Caspase-11^−/−^* mice had significantly decreased bacterial CFUs compared to the wildtype. The increased bacteria load in *Asc^−/−^* mice is consistent with the increased morbidity and reduced inflammation in Figure 1 and Figure 2, and further illustrates the critical role of ASC in the host response to *B. abortus*. Likewise, these *Caspase-11^−/−^* data suggest a significant, but variable, role in controlling the *B. abortus* bacteria burden that does not appear to impact the overall host morbidity or inflammation.

### 2.4. B. abortus Initiates a Weak Inflammasome-Mediated Inflammatory Cytokine Response

**Figure 4:**
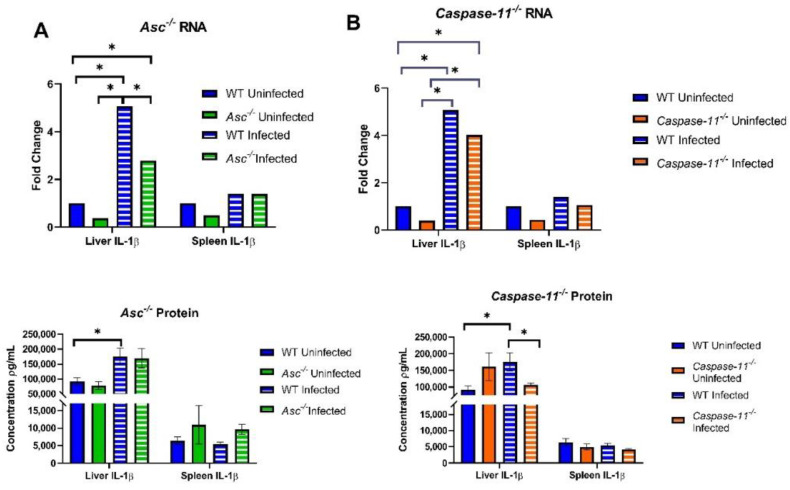
Inflammatory signaling in the liver and spleen of *Asc^−/−^* and *Caspase-11^−/−^* mice. Three d.p.i. livers and spleens of 34 C57BL/6 WT (*n* = 11 U, 23 I), (**A**) 20 *Asc^−/−^* (*n* = 6 U, 14 I), and (**B**) 27 *Caspase-11^−/−^* (*n* = 8 U, 19 I) mice were homogenized and analyzed for their *Il1β* RNA fold change using RT-PCR and IL-1β protein concentration through ELISA. * *p* < 0.05.

Inflammasome activation results in the cleavage and processing of IL-1β and IL-18. To evaluate the generation of pro-IL-1β, so-called “Signal 1”, at the transcript level, we conducted quantitative real-time PCR in liver and spleen homogenates (Figure 4). We observed significantly increased fold changes in *Il1β* in the livers of *Asc^−/−^* and wildtype mice infected with *B. abortus* versus the uninfected mice (Figure 4A). Within infected mouse groups in the liver, we also found that infected *Asc^−/−^* mice had a significantly decreased fold change of *Il1β* compared to wildtype mice (Figure 4A). However, when assessing the total IL-1β (uncleaved and cleaved) protein, we did not see a significant difference between the infected wildtype and *Asc^−/−^* mice.

In experiments with *Caspase-11^−/−^* mice, we also found an elevated RNA fold change between the infected and uninfected groups (Figure 4B), but there was no significant difference between the infected wildtype and *Caspase-11^−/−^* mice for *Il1β* transcription (Figure 4B). However, there was a minimal, but statistically significant, decrease in IL-1β protein in the livers from *Caspase-11^−/−^* mice compared to the wildtype animals.

### 2.5. B. abortus Infection Attenuated IL-1β and Induced a Strong ASC-Dependent Pyroptosis Response in Macrophages

**Figure 5:**
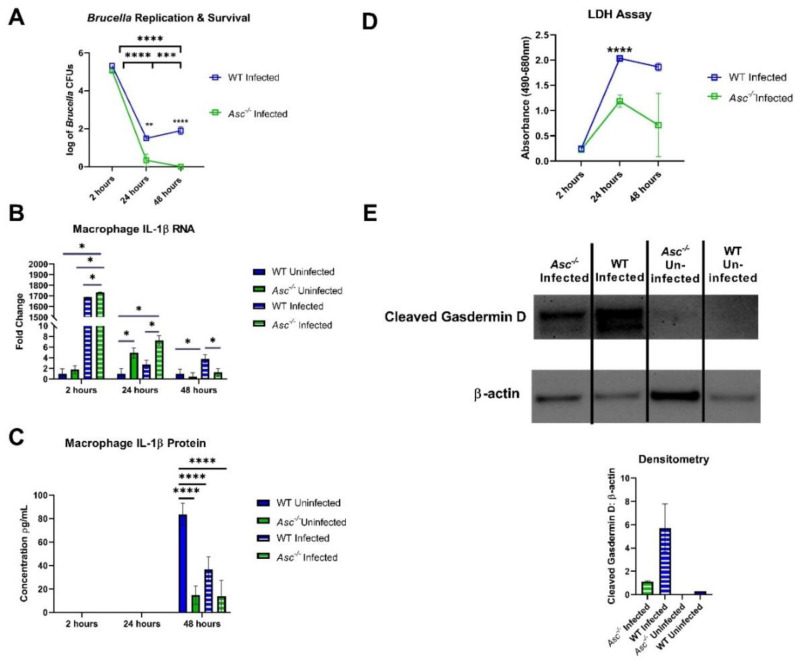
ASC-dependent IL-1β generation and pyroptosis following *B. abortus* infection in BMDMs. Bone marrow-derived macrophages (BMDMs) (500,000 cells per well, *n* = 3 per group) were harvested from C57BL/6 WT and *Asc^−/−^* mice and challenged with a MOI 100:1 (10^7^ CFUs) of *Brucella abortus.* (**A**) BMDMs were measured for CFUs for a 48 h period post-challenge. (**B**) BMDMs were measured for the *Il1β* fold change in RNA through RT-PCR. (**C**) BMDMs were analyzed for IL-1β protein in the supernatant using an ELISA. (**D**) Lactate Dehydrogenase (LDH) was measured through spectrophotometry through a 48 h time period post-challenge. (**E**) BMDMs were lysed at 24 h post-challenge and used for Western blot analysis. BMDM protein (20μg) was probed for cleaved gasdermin D and β-actin as a control for the protein amount. Invitrogen IBright Analysis was used to determine the density ratio between the cleaved gasdermin D over β-actin. * *p* < 0.05, *** *p* < 0.001, **** *p* < 0.0001.

Due to the significant phenotype observed in the *Asc^−/−^* mice, we next sought to better define the underlying mechanism using ex vivo bone marrow-derived macrophages (BMDMs). Intracellular bacterial replication and survival was evaluated over a 48 h period in *B. abortus*-infected *Asc^−/−^* and wildtype BMDMs. Under these conditions, we observed a significant decrease in *B. abortus* growth in the *Asc^−/−^* macrophages compared with the wildtype BMDMs after 24 h (Figure 5A). By 48 h, the *B. abortus* replication and survival was no longer detectable, while increasing in the wildtype BMDMs. These results were unexpected based on our in vivo findings and suggest a possible disconnect between pathogen clearance, inflammasome function, and pyroptosis in BMDMs.

We next measured the RNA fold change and protein concentration of IL-1β (Figure 5B,C). Within 2 h of *B. abortus* exposure, we observed elevated *Il1β* transcription in the infected wildtype and *Asc^−/−^* macrophages. Transcription was statistically significant, but only slightly higher, in the *Asc^−/−^* cells compared to the wildtype BMDMs (Figure 5B). At 24 h and 48 h, we observed a significant decrease in *Il1β* transcription in both groups of infected mice, with significantly more *Il1β* in the *Asc^−/−^* BMDMs at 24 h compared to the wildtype (Figure 5B). Complementing the transcription data, we also evaluated the IL-1β protein levels in the cell supernatant using ELISA (Figure 5C). These levels were significantly attenuated in the *B. abortus*-infected cells (Figure 5C). We also observed a significant decrease in IL-1β in the *Asc^−/−^* BMDMs under all conditions (Figure 5C), emphasizing that IL-1β processing is ASC-dependent.

To further expand upon our cell death findings, we conducted a LDH assay in our *Asc^−/−^* and wildtype macrophages to quantify the lactate dehydrogenase enzyme released from dead cells (Figure 5D). We found that, starting at 24 h, there was significantly higher cell death occurring in our wildtype cells compared to the *Asc^−/−^* macrophages. To further define the mechanism of cell death, we evaluated pyroptosis by determining gasdermin D cleavage using Western blot. We found a significant increase in cleaved gasdermin D 24 h post-infection in the wildtype BMDMs compared to the significantly reduced levels observed in the *Asc^−/−^* macrophages (Figure 5E). This was confirmed using densitometry (Figure 5E).

### 2.6. B. abortus gDNA is a Potent PAMP Associated with ASC-Dependent Canonical Inflammasome Signaling

**Figure 6:**
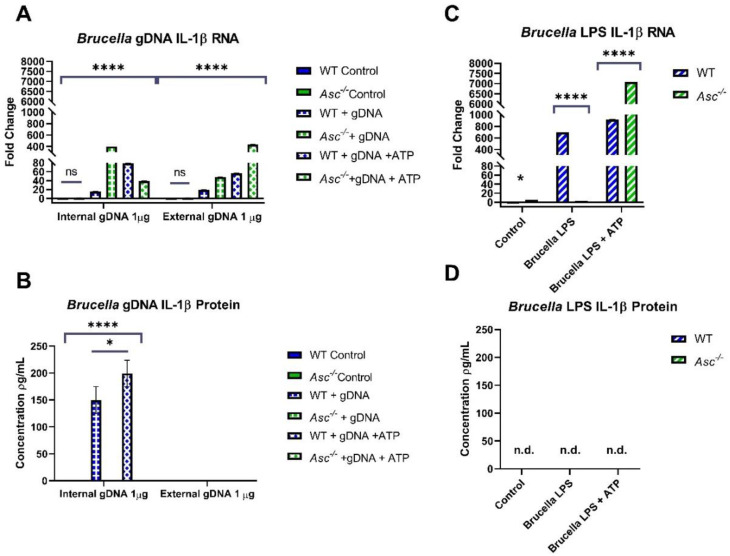
*B. abortus* gDNA is a potent PAMP and induces ASC-dependent IL-1β production. Bone marrow-derived macrophages (BMDMs) (500,000 cells per well, *n* = 2 per group) were harvested from C57BL/6 WT and *Asc^−/−^* mice. (**A**,**B**) BMDMs were challenged with 1 μg of gDNA (2μg/mL) externally in media or transfected internally with 300 μM of ATP and harvested after 24 h. BMDMs were analyzed for the IL-1β (**A**) RNA fold change through RT-PCR and (**B**) protein concentration through ELISA. (**C**,**D**) BMDMs were challenged with 1 μg/mL of *Brucella* LPS externally to the media with 300 μM of ATP and harvested after 8 h. BMDMs were analyzed for the IL-1β (**C**) RNA fold change through RT-PCR and (**D**) protein concentration through ELISA. Note that there was no detectable (*n*.d.) protein concentration in (**D**). * *p* < 0.05, **** *p* < 0.0001.

To further define inflammasome activation following *B. abortus* infection, we next evaluated the potential pathogen-associated molecular patterns (PAMPs), focusing on bacterial gDNA. We challenged macrophages with 1 μg of gDNA (2 μg/mL) both externally, by adding gDNA to the cell media, and internally through the Lipofectamine 3000 reagent (Figure 6A). We also added 300 μM of ATP to augment the IL-1β release. *B. abortus* gDNA induced a significant increase in the *Il1β* gene transcription 24 h post challenge, following either extracellular or intracellular challenge (Figure 6A). We observed significant increases in *Il1β* transcription under several different conditions in gDNA-challenged *Asc^−/−^* macrophages compared to the wildtype cells. ELISA assessments revealed that IL-1β protein was only released into the supernatant following internal gDNA stimulation in wildtype cells (Figure 6B). This was highly dependent on ASC. The *B. abortus* gDNA challenge resulted in significant increases in IL-1β in the wildtype cells, whereas the levels were below the level of detection in the *Asc^−/−^* cells (Figure 6B). Together, these data confirm *B. abortus* gDNA as a potent PAMP and suggest that its recognition by the canonical inflammasome, in an ASC-dependent mechanism, underlies host defense. In addition to gDNA, we also evaluated the ability of the canonical inflammasome to recognize *B. abortus* LPS (1 μg/mL externally). The fold change in *Il1β* RNA at 8 h post challenge indicated that there was elevated transcription in wildtype macrophages over *Asc^−/−^* cells (Figure 6C). However, there was no IL-1β protein signaling for *Brucella* LPS (Figure 6D).

## 3. Discussion

In this report, we assessed inflammasome activation following *Brucella abortus* infection. During canonical inflammasome activation, a pathogen is sensed by a NLR or ALR pattern recognition receptor and forms a multi-protein complex with the binding protein ASC and caspase-1. This process can initiate the cleavage of IL-1β and IL-18 in addition to pyroptosis [8]. While highly related to the canonical inflammasome pathway, non-canonical inflammasome activation is often more closely associated with the promotion of pyroptosis and the activation of caspase-11 [26]. Together, our in vivo data reveal a more robust phenotype in the *Asc^−/−^* mice compared to the *Caspase-11^−/−^* animals, suggesting that the canonical inflammasome plays a significantly greater role in host pathogen defense following *B. abortus* infection. ASC and, by extension, the canonical inflammasome promotes survival, augments inflammation, and attenuates bacterial load *in vivo*. This result is consistent with others in the field that have used myriad of other inflammasome knockout models [5,6,7,27]. However, there are certainly exceptions to these findings. For example, Gomes et al. (2103) recently reported no inflammasome knockout mice (*n* = 5 per group) exhibited mortality under their experimental conditions [6]. One difference to note is that our study utilized a much larger sample size per group (*n* = 19). Because many of our animals recovered, it is certainly possible that greater power through that increased sample size is necessary to better reflect the mortality data for the *Asc^−/−^* animals. Ultimately, our comparison between *Asc^−/−^* and *Caspase-11^−/−^* mice expands upon the findings of many of these prior studies and provides a more direct assessment of the pathobiological effects of the canonical and non-canonical inflammasome in *B. abortus* host defense.

Although non-canonical signaling through the *Brucella* LPS activation of caspase-11-mediated pyroptosis has been previously indicated [28], we found inconsistent results in inflammasome activation under our conditions. In *Caspase-11^−/−^* mice, we found no loss in survival or morbidity, no significant inflammation through histopathology scoring, and no protective role in promoting inflammatory signaling. Previous studies using *Caspase-11^−/−^* mice mimicked our non-significant results in bacterial load at 3 days post-infection and only found significant bacterial load 1–2 weeks after infection [28]. Although we did not assess the bacterial load or inflammatory signaling after 3 d.p.i in this report, we did assess the morbidity and mortality of *Caspase-11^−/−^* mice over 3 weeks, in which knockouts exhibited no decrease in morbidity or loss in survival. This indicates that caspase-11 may play a small but relatively insignificant role in promoting pyroptosis that perhaps may be slightly amplified 1–2 weeks after *Brucella* infection. Additionally, these studies utilized immune cell priming in macrophages with PAMPs, such as *E. coli* LPS and Pam3CSK4, and subsequently observed high cytokine signaling in their challenge [28]. Contrasting this data, our results indicated no IL-1β protein response to *Brucella* LPS stimulation in unprimed macrophages. These data suggest that the immune adjuvants directly impact the activation of caspase-11 and that the role of non-canonical inflammasome in host defense against *B. abortus* is minimal in the absence of macrophage priming.

Previously, pyroptosis had only been attributed to non-canonical inflammasome activation by *Brucella* spp. [6,28]. This is further confirmed in this report through the presence of cleaved gasdermin D bands in *Asc^−/−^* macrophages, indicating that ASC-independent pyroptosis occurs in response to *Brucella*. However, our research demonstrates that the removal of ASC-dependent inflammasome activation significantly decreases the activation of gasdermin D to form pyroptotic pores. As described in the literature, ASC specks serve as recruitment factors for procaspase-1 through the polymerization of its caspase activation recruitment domain (CARD). Caspase-1 only becomes activated through this process during ASC-dependent inflammasome formation [29]. In turn, caspase-1 cleaves gasdermin D, which has been identified as the most significant gene initiating caspase-1 induced pyroptosis, and initiates inflammatory cell death [30]. Our results are consistent with this ASC-mediated pathway of the capsase-1 activation of pyroptosis that is dependent on gasdermin D cleavage. To date, we know that *Brucella* initiates the caspase-1 and -11 activation of pyroptosis in joints of animal models [31]. Additionally, pyroptosis is activated by gDNA in dendritic cells [5]. Our data supports a model where both caspase-1 and -11 promote pyroptosis, and where gDNA from *B. abortus* functions as a robust PAMP that specifically activates the canonical inflammasome, driving ASC-dependent inflammation and pyroptosis.

Previous literature indicates that the role of pyroptosis in brucellosis serves to restrict *Brucella* growth in macrophages of the joints and control infection [31]. Our findings are consistent with this previous study. Under our conditions, the ASC-mediated initiation of pyroptosis appears to ensure mouse survival, immune cell recruitment, and inflammatory signaling. However, we should also point out the bacteria clearance and IL-1β data in the BMDM studies (Figure 5A,C). In Figure 5A, these data would suggest that the lack of ASC and canonical inflammasome signaling actually improved the bacteria clearance from these BMDMs, despite having reduced IL-1β and pyroptosis. While these data seem to conflict with each other, several recent studies have reported similar findings for other bacterial pathogens. For example, *Citrobacter rodentium* infection results in significant osmotic changes in targeted cells that can augment inflammasome signaling [32]. However, the clearance of the pathogen itself appears to be independent of the inflammasome and the ASC modulation of inflammation and pyroptosis [33]. Thus, it is possible that a similar mechanism is associated here with *B. abortus*. Looking at the IL-1β graph in Figure 5C, it suggests that *B. abortus* infection in macrophages suppresses IL-1β production from wildtype cells. It is possible that the attenuation of total IL-1β in this figure may be due to the subversion of TLR signaling generating pro- IL-1β. Many studies have shown *Brucella* subversion of TLR signaling through proteins such as Tcbp, which leads to decreased proinflammatory cytokine expression [10]. Additionally, *Brucella* microRNAs can lead to the downregulation of the mRNA and protein expression of innate immune PRRs [34]. These mechanisms likely did not occur in our gDNA studies as there was no inclusion of these immunosuppressive proteins or production of inhibitory microRNAs. Therefore, it is possible that the full *Brucella* bacterium may be utilizing these methods of immunosuppression to contribute to decreased total IL-1β.

Our data suggests that ASC and the canonical inflammasome contribute to host defense in response to *B. abortus*. These results are consistent with several others in the field and provide additional insight into host defense against this highly intriguing pathogen. Currently, brucellosis is having a significantly negative impact on a growing number of human populations worldwide. Therefore, it is essential that we expand our understanding of the underlying disease mechanisms and host immune response to *B. abortus*. This finding that the canonical inflammasome plays a dominate role in driving the host innate immune response and pyroptosis following the sensing of gDNA is an encouraging discovery that may contribute to the development of future therapeutics or strategic approaches to combat this disease and its underlying pathogen.

## 4. Materials and Methods

### 4.1. Bacterial Strains and Growth Conditions

*Brucella abortus* 2308 was routinely grown on Schaedler blood agar (SBA), which is composed of Schaedler agar (BD, Franklin Lakes, NJ, USA) containing 5% defibrinated bovine blood (Quad Five, Ryegate, MT, USA). All work with live *Brucella* strains was performed in a biosafety level 3 (BSL3) facility. Animal work conducted in ABSL3 conditions was conducted **under IACUC protocol** # **14-055** at Virginia Tech following the ethical standards of animal use in research.

### 4.2. In Vivo Brucella Studies

C57BL/6 WT mice (*n* = 26), *Asc^−/−^* mice (C57BL/6 background; *n* = 19), and *Caspase-11^−/−^* (C57BL/6 background; *n* = 19) mice were inoculated intraperitoneally with 1 × 10^5^ CFU of *Brucella abortus*. The percent weight change was measured each day post-*Brucella* inoculation to determine morbidity warranting euthanasia (>20%) following the IACUC protocol.

Additionally, 20 canonical knockout (*Asc^−/−^*, *n =* 6 U, 14 I), 27 non-canonical (*Caspase-11^−/−^*, *n* = 8 U, 19 I), and 34 C57BL/6 WT (*n* = 11 U, 23 I) mice were euthanized at 3 days post infection and harvested for the liver and spleen. Both organs were sectioned into three equal parts. The first section of both organs was taken for histopathology analysis to determine the scoring of extramedullary hematopoiesis (EMH) and inflammation. The remaining sections were homogenized in 1×PBS and analyzed for the number of CFUs per gram and the RNA and protein concentrations of IL-1β. RNA was isolated from the liver and spleen with TRIzol reagents (Invitrogen) followed by ethanol precipitation. Genomic DNA was removed with DNase I, and samples were cleaned using phenol-chloroform extractions and precipitated with ethanol. RNA samples were then resuspended in nuclease-free H_2_O, and the purity of samples was checked with a NanoDrop 1000 spectrophotometer (ThermoFisher).

After isolation, RNA was converted into 1 μg cDNA through a High-Capacity cDNA Reverse Transcription Kit (ThermoFisher). This cDNA was analyzed for IL-1β by RT-qPCR (40× cycles) using Taqman Fast MasterMix (ThermoFisher). Protein was determined through a sandwich ELISA kit (R&D systems).

### 4.3. Bone Marrow-Derived Macrophage (BMDM) Isolation

Bone marrow-derived macrophages (BMDMs) were derived from the bone marrow of C57BL/6 WT and *Asc^−/−^* mice under the **IACUC protocol #18-104** at Virginia Tech. Two adult mice from each group were sacrificed using CO_2_ fixation followed by cervical dislocation. Bone marrow was extracted from the tibias and femora of the mice. Cells were cultured in non-TC culture dishes in our formulation of culture media (Dulbecco’s Modified Eagle Medium (DMEM) (ThermoFisher) containing 10% Fetal Bovine Serum (FBS), 1% penicillin/streptomycin, 1% nonessential amino acids, and 20% L929 conditioned media [35]). These culture dishes were incubated at 37 °C with 5% CO_2_. After 6–7 days of culture, these cells had differentiated into macrophages. BMDMs were collected from the bottom of the plates through a cold 1× PBS solution containing 5 mM of EDTA. After 1 h on ice, we checked for macrophage detachment via microscope. Macrophages were collected and seeded at 500,000 cells/well in 24-well plates in media without antibiotics and left to adhere overnight in culture media.

### 4.4. Live Brucella Challenge in BMDMs

Macrophages were infected with a MOI 100:1 (10^7^ CFUs/5 × 10^5^ BMDMs) with *B. abortus* 2308. At the 2, 24, and 48 h time points, intracellular *B. abortus* was determined through a gentamicin protection assay [36]. At 2 h, the infected macrophages were treated with gentamicin (50 μg/mL) for 1 h. Macrophages were then lysed with 0.1% deoxycholate in PBS, and serial dilutions were plated on Schaedler blood agar (SBA) containing 5% bovine blood (Quad Five). For the 24 and 48 h time points, macrophages were washed with PBS and fresh cell culture medium containing gentamicin (20 μg/mL) was added to the macrophages. At the indicated time points, macrophages were lysed, and serial dilutions were plated on SBA in triplicates.

The supernatant of BMDMs was sterile-filtered for later IL-1β protein analysis through a sandwich ELISA kit (R&D systems). Macrophages at each time point were lysed with 0.1% deoxycholate and isolated for RNA. After isolation, RNA was converted into 0.5μg cDNA through a High-Capacity cDNA Reverse Transcription Kit (ThermoFisher). This cDNA was analyzed for IL-1β by RT-qPCR (40× cycles) using Taqman Fast MasterMix (ThermoFisher).

### 4.5. LDH Assay

Macrophages were infected with a MOI 100:1 (10^7^ CFUs/5 × 10^5^ BMDMs) with *B. abortus.* At the 2, 24, and 48 h time points, extracellular *B. abortus* was killed through gentamicin (50 µg/mL). The supernatant of BMDMs was collected and centrifuged at 1000× *g* for 10 min to remove cell debris. The remaining supernatant (50 μL) was used for the CyQUANT™ LDH Cytotoxicity Assay kit (Invitrogen) and read at a corrected absorbance of 490–680 nm.

### 4.6. Brucella PAMP Isolation

#### 4.6.1. *Brucella* gDNA

*B. abortus* gDNA was isolated from the 2308 strain by phenol:chloroform extraction. Approximately 3 mL of an overnight culture of *B. abortus* was pelleted by centrifugation. The pellet was resuspended in 200 μL of 0.04 M sodium acetate, 200 μL of 10% SDS, and 600 μL of TRIzol. A total of 250 μL of chloroform was added to this mixture in a Phase Lock tube (5PRIME) and centrifuged at 20,000× *g* (max speed) for 2 min. A second chloroform wash was performed in a Phase Lock tube. The resulting aqueous layer is then removed and added to 1 mL of 100% ethanol. DNA precipitation is carried out from this point.

#### 4.6.2. *Brucella* LPS

*B. abortus* LPS was isolated from the *B. abortus* 2308 using hot-phenol extraction, as described previously [31]. Bacteria were killed with ethanol: acetone, and the cells were recovered by centrifugation. The pellet was suspended in deionized water at 66 °C and then mixed with 90% phenol *w/v* that was heated to 66 °C. After stirring for 20 min, the suspension was chilled on ice. The solution was then subjected to centrifugation (15 min at 13,000× *g*). The phenol layer was aspirated, filtered through a Whatman #1 filter, and the LPS was precipitated with methanol containing 1% methanol saturated with sodium acetate. Following incubation at 4 °C for 1 h, the mixture was subjected to centrifugation (10,000× *g* for 10 min). The precipitate was stirred with deionized water for 12 h at 4 °C. Following centrifugation (10,000× *g* for 10 min), the supernatant was precipitated with trichloroacetic acid, and the resulting supernatant following centrifugation (10,000× *g* for 10 min) was dialyzed with deionized water and stored at −20 °C. The concentration of LPS was determined through the Pierce™ Chromogenic Endotoxin Quant Kit (ThermoFisher).

### 4.7. Brucella PAMP Challenge

After PAMP isolation, *B. abortus* gDNA (2 μg/mL) and LPS (1 μg/mL) were introduced to BMDMs. gDNA was introduced both intracellularly, through the Lipofectamine 3000 Transfection Reagent (Invitrogen), and extracellularly in media. LPS was only introduced extracellularly. Timepoints for this challenge included 24 h for gDNA and 8 h for LPS post-challenge. Samples were run with and without the priming of 300 μM of ATP 45 min before each time point to stimulate IL-1β protein release after transcription. At each time point, supernatant was collected from each well and centrifuged at 1000× *g* for 10 min to remove cell debris. The supernatant was later used for protein quantification using an IL-1β sandwich ELISA (R&D Systems). Macrophages were lysed with 200 μL of TRIzol Reagent (Invitrogen) and followed the TRIzol RNA isolation protocol. After isolation, RNA was converted into 1 μg of cDNA through a High-Capacity cDNA Reverse Transcription Kit (ThermoFisher). This cDNA was analyzed for IL-1β by RT-qPCR (40× cycles) using the Taqman Fast MasterMix (ThermoFisher).

### 4.8. Western Blot

Macrophages from live *Brucella* challenge were lysed using a sodium lysis buffer (0.3% SDS, 200 mM dithiothreitol, 22 mM Tris-base, and 28 mM Tris-HCl pH 8.0). Samples were then boiled for a period of 1 h, vortexing the samples every 10 min. Samples were frozen at −20 °C until use. Prior to running the gel, samples were sonicated for 10 s each. Protein quantification was determined using the Pierce™ Detergent Compatible Bradford Assay Kit (ThermoFisher). Samples (20 μg of protein) were heated at 97 °C with a reducing buffer for 7 min and run on pre-cast Bolt™ 4 to 12%, Bis-Tris, 1.0 mm, Mini Protein Gel, 10-well gels (Invitrogen) for 45 min at 165 V with a 1× Micro Extraction Packet Sorbent (MEPS) buffer (ThermoFisher). Gel was transferred onto a polyvinylidene difluoride (PVDF) membrane using a transfer chamber with transfer buffer (20% methanol in 1× Tris Glycine (TGE)). The membrane was then blocked in 5% milk for 1 h, and then incubated overnight with a cleaved gasdermin D rabbit antibody (diluted 1:1000, Cell Signaling #36425S). Membrane was washed in Tris-buffered saline with Tween 20 (1× TBST) 4× for 15 min each and then blocked with goat anti-rabbit IgG antibody conjugated with horseradish peroxidase (HRP) (diluted 1:2000, Cell Signaling #7074) for 1 h. Membrane was washed again with 1× TBST 4× for 15 min each and then imaged using West Pico Substrate for imaging (ThermoFisher). Gels were imaged using an IBright CL1500 imaging machine. Sample bands were normalized using the β-actin rabbit antibody (Cell Signaling #4970) using the same protocol above. The density of bands was calculated using the IBright Analysis software (ThermoFisher) to calculate a cleaved gasdermin D/β-actin ratio.

### 4.9. Graphing and Statistical Analyses

All the figures and statistical analyses were generated in GraphPad 8.4.3 (Prism). Statistical tests included two-way ANOVAs, using the Tukey or Sidak post-hoc tests, and two sample *t*-tests when appropriate. All the data are contained within the article.

## References

[1] Corbel M.J. (1997). Brucellosis: An overview. Emerg. Infect. Dis..

[2] Lapaque N., Moriyon I., Moreno E., Gorvel J.P. (2005). *Brucella* lipopolysaccharide acts as a virulence factor. Curr. Opin. Microbiol..

[3] De Figueiredo P., Ficht T.A., Rice-Ficht A., Rossetti C.A., Adams L.G. (2015). Pathogenesis and immunobiology of brucellosis: Review of *Brucella*–Host Interactions. Am. J. Pathol..

[4] Janeway C.A. (1989). Approaching the asymptote? Evolution and revolution in immunology. Cold Spring Harb. Symp. Quant. Biol..

[5] Franco M.M.S.C., Marim F.M., Alves-Silva J., Cerqueira D., Rungue M., Tavares I.P., Oliveira S.C. (2019). AIM2 senses *Brucella abortus* DNA in dendritic cells to induce IL-1β secretion, pyroptosis and resistance to bacterial infection in mice. Microbes Infect..

[6] Gomes M.T.R., Campos P.C., Oliveira F.S., Corsetti P.P., Bortoluci K.R., Cunha L.D., Zamboni D.S., Oliveira S.C. (2013). Critical role of ASC inflammasomes and bacterial type IV secretion system in caspase-1 activation and host innate resistance to *Brucella abortus* infection. J. Immunol..

[7] Marim F.M., Franco M.M.C., Gomes M.T.R., Miraglia M.C., Giambartolomei G.H., Oliveira S.C. (2017). The role of NLRP3 and AIM2 in inflammasome activation during *Brucella abortus* infection. Semin. Immunopathol..

[8] Coutermarsh-Ott S., Eden K., Allen I.C. (2016). Beyond the inflammasome: Regulatory NOD-like receptor modulation of the host immune response following virus exposure. J. Gen. Virol..

[9] Tupik J.D., Nagai-Singer M.A., Allen I.C. (2020). To protect or adversely affect? The dichotomous role of the NLRP1 inflammasome in human disease. Mol. Asp. Med..

[10] Oliveira S.C., de Oliveira F.S., Macedo G.C., de Almeida L.A., Carvalho N.B. (2008). The role of innate immune receptors in the control of *Brucella abortus* infection: Toll-like receptors and beyond. Microbes Infect..

[11] Campos M.A., Rosinha G.M., Almeida I.C., Salgueiro X.S., Jarvis B.W., Splitter G.A., Qureshi N., Bruna-Romero O., Gazzinelli R.T., Oliveira S.C. (2004). Role of Toll-like receptor 4 in induction of cell-mediated immunity and resistance to *Brucella abortus* infection in mice. Infect. Immun..

[12] De Almeida L.A., Macedo G.C., Marinho F.A., Gomes M.T., Corsetti P.P., Silva A.M., Cassataro J., Giambartolomei G.H., Oliveira S.C. (2013). Toll-like receptor 6 plays an important role in host innate resistance to *Brucella abortus* infection in mice. Infect. Immun..

[13] Ferrero M.C., Hielpos M.S., Carvalho N.B., Barrionuevo P., Corsetti P.P., Giambartolomei G.H., Oliveira S.C., Baldi P.C. (2014). Key role of Toll-like receptor 2 in the inflammatory response and major histocompatibility complex class II downregulation in *Brucella abortus*-infected alveolar macrophages. Infect. Immun..

[14] Campos P.C., Gomes M.T.R., Guimarães E.S., Guimarães G., Oliveira S.C. (2017). TLR7 and TLR3 sense *Brucella abortus* RNA to induce proinflammatory cytokine production but they are dispensable for host control of infection. Front. Immunol..

[15] Macedo G.C., Magnani D.M., Carvalho N.B., Bruna-Romero O., Gazzinelli R.T., Oliveira S.C. (2008). Central role of MyD88-dependent dendritic cell maturation and proinflammatory cytokine production to control *Brucella abortus* infection. J. Immunol..

[16] De Almeida L.A., Carvalho N.B., Oliveira F.S., Lacerda T.L.S., Vasconcelos A.C., Nogueira L., Bafica A., Silva A.M., Oliveira S.C. (2011). MyD88 and STING signaling pathways are required for IRF3-mediated IFN-β induction in response to *Brucella abortus* infection. PLoS ONE.

[17] Hornung V., Latz E. (2010). Critical functions of priming and lysosomal damage for NLRP3 activation. Eur. J. Immunol..

[18] Miao E.A., Andersen-Nissen E., Warren S.E., Aderem A. (2007). TLR5 and Ipaf: Dual sensors of bacterial flagellin in the innate immune system. Semin. Immunopathol..

[19] Martinon F., Burns K., Tschopp J. (2002). The inflammasome: A molecular platform triggering activation of inflammatory caspases and processing of proIL-beta. Mol. Cell.

[20] Agostini L., Martinon F., Burns K., McDermott M.F., Hawkins P.N., Tschopp J. (2004). NALP3 forms an IL-1beta-processing inflammasome with increased activity in Muckle-Wells autoinflammatory disorder. Immunity.

[21] Davis B.K., Roberts R.A., Huang M.T., Willingham S.B., Conti B.J., Brickey W.J., Barker B.R., Kwan M., Taxman D.J., Accavitti-Loper M.A. (2011). Cutting edge: NLRC5-dependent activation of the inflammasome. J. Immunol..

[22] Wlodarska M., Thaiss C.A., Nowarski R., Henao-Mejia J., Zhang J.P., Brown E.M., Frankel G., Levy M., Katz M.N., Philbrick W.M. (2014). NLRP6 inflammasome orchestrates the colonic host-microbial interface by regulating goblet cell mucus secretion. Cell.

[23] Vanaja S.K., Rathinam V.A.K., Fitzgerald K.A. (2015). Mechanisms of inflammasome activation: Recent advances and novel insights. Trends Cell Biol..

[24] Shi J., Gao W., Shao F. (2017). Pyroptosis: Gasdermin-mediated programmed necrotic cell death. Trends Biochem. Sci..

[25] Franco M.M.C., Marim F., Guimarães E.S., Assis N.R., Cerqueira D.M., Alves-Silva J., Harms J., Splitter G., Smith J., Kanneganti T.-D. (2018). *Brucella abortus* triggers a cGAS-independent STING pathway to induce host protection that involves guanylate-binding proteins and inflammasome activation. J. Immunol..

[26] Yi Y.-S. (2018). Regulatory Roles of the Caspase-11 Non-Canonical Inflammasome in Inflammatory Diseases. Immune Netw..

[27] Hielpos M.S., Fernández A.G., Falivene J., Alonso Paiva I.M., Muñoz González F., Ferrero M.C., Campos P.C., Vieira A.T., Oliveira S.C., Baldi P.C. (2018). IL-1R and inflammasomes mediate early pulmonary protective mechanisms in respiratory *Brucella abortus* infection. Front. Cell. Infect. Microbiol..

[28] Cerqueira D.M., Gomes M.T.R., Silva A.L., Rungue M., Assis N.R., Guimarães E.S., Morais S.B., Broz P., Zamboni D.S., Oliveira S.C. (2018). Guanylate-binding protein 5 licenses caspase-11 for Gasdermin-D mediated host resistance to *Brucella abortus* infection. PLoS Pathog..

[29] Lu A., Magupalli V.G., Ruan J., Yin Q., Atianand M.K., Vos M.R., Schröder G.F., Fitzgerald K.A., Wu H., Egelman E.H. (2014). Unified polymerization mechanism for the assembly of ASC-dependent inflammasomes. Cell.

[30] Broz P. (2015). Caspase target drives pyroptosis. Nature.

[31] Lacey C.A., Mitchell W.J., Dadelahi A.S., Skyberg J.A. (2018). Caspase-1 and caspase-11 mediate pyroptosis, inflammation, and control of *Brucella* joint infection. Infect. Immun..

[32] Liu Z., Zaki M.H., Vogel P., Gurung P., Finlay B.B., Deng W., Lamkanfi M., Kanneganti T.D. (2012). Role of inflammasomes in host defense against *Citrobacter rodentium* infection. J. Biol. Chem..

[33] Armstrong H., Bording-Jorgensen M., Chan R., Wine E. (2019). Nigericin Promotes NLRP3-Independent Bacterial Killing in Macrophages. Front. Immunol..

[34] Khan M., Harms J.S., Liu Y., Eickhoff J., Tan J.W., Hu T., Cai F., Guimaraes E., Oliveira S.C., Dahl R. (2020). *Brucella* suppress STING expression via miR-24 to enhance infection. PLoS Pathog..

[35] Englen M.D., Valdez Y.E., Lehnert N.M., Lehnert B.E. (1995). Granulocyte/macrophage colony-stimulating factor is expressed and secreted in cultures of murine L929 cells. J. Immunol. Methods.

[36] Sharma A., Puhar A. (2019). Gentamicin Protection Assay to Determine the Number of Intracellular Bacteria during Infection of Human TC7 Intestinal Epithelial Cells by Shigella flexneri. Bio-Protocol.

